# Effect of Magnesium and Temperature on the Accelerated Carbonation Progress of β-Dicalcium Silicate

**DOI:** 10.3390/ma18102232

**Published:** 2025-05-12

**Authors:** Binbin Fu, Chaoran Wang, Dan Wang

**Affiliations:** 1 State Key Laboratory of Marine Resource Utilization in South China Sea, Hainan University, Haikou 570228, China; fubinbin0214@163.com; 2 College of Materials Science and Engineering, Hainan University, Haikou 570228, China; 3 School of Architectural Engineering, Jiyuan Vocational and Technical College, Jiyuan 459000, China; 15993700395@163.com

**Keywords:** β-dicalcium silicate, carbonation, aragonite, temperature, magnesium

## Abstract

This study investigates the impact of different temperatures and initial Mg^2+^/Ca^2+^ molar ratios in the solution on the wet-accelerated carbonation of β-dicalcium silicate (β-C_2_S). The x-ray diffraction (XRD), fourier transform infrared spectroscopy (FT-IR), thermogravimetric analyzer (TGA), and field emission scanning electron microscopy (FE-SEM) analysis results indicated that temperature and the Mg^2+^/Ca^2+^ molar ratio are key factors in the nucleation of aragonite. Aragonite formed at a temperature above 60 °C, and the high temperature promoted the crystallinity of needle-like aragonite with a length of 1–6 μm and a diameter of ~1 μm. Moreover, 80 °C was the most favorable temperature for the formation of aragonite with a large aspect ratio in the carbonation system of β-C_2_S. Mg^2+^ had a significant effect on inhibiting the transformation of aragonite to calcite and promoting the stability of aragonite. Aragonite became the dominant CaCO_3_ polymorph instead of calcite when the Mg^2+^/Ca^2+^ molar ratio was above 1.0, and pure aragonite-style calcium carbonate was formed at a Mg^2+^/Ca^2+^ molar ratio of 1.5.

## 1. Introduction

With the continuous developments in industrialization, the concentration of CO_2_ in the atmosphere continues to rise. It was reported that approximately 36 billion metric tons of CO_2_ are emitted globally every year, and this number continues to rise [1].

The cement industry accounts for about 8% of global CO_2_ emissions [2]. The reasons include both electric energy consumption and the decomposition process of the main raw material, limestone. Therefore, the world is currently facing an enormous environmental issue. To mitigate global warming and other related climate change due to CO_2_ emissions, the technology of CO_2_ sequestration in cement-based materials is attracting more attention [3,4,5]. Mineral carbonates such as CaCO_3_ or MgCO_3_ are the most thermodynamically stable forms of carbon, and once carbon dioxide is converted into carbonates, long-term storage can be achieved [6]. Hence, the accelerated carbonation of cement materials has great potential for industrial applications, which is an important technology to reduce carbon footprints [7,8].

As a kind of cement-based material, steel slag waste used as a building material is a promising approach to significantly enhance its utilization rate. However, the complex chemical composition and low cementitious property of steel slag are the main challenges in its application [9,10]. Due to the high carbon sequestration ability of steel slag, attempts have been made to chemically capture and store CO_2_ by accelerating its carbonation [11,12,13]. β-C_2_S belongs to the monoclinic crystal system and features an island silicate structure. Within its structural framework, the [SiO_4_] tetrahedra structural units are interconnected via [CaO_x_] polyhedra to form a three-dimensional spatial architecture [14]. This structural configuration endows β-C_2_S with moderate hydration reactivity and high carbonation activity, making it the key cementitious phase in steel slag (constituting ~20 wt.%) [15]. In contrast, while γ-dicalcium silicate (γ-C_2_S) theoretically exhibits higher carbonation reactivity [16], it demonstrates extremely low hydration activity and is rarely found in industrial by-products such as steel slag. Consequently, studying the carbonation behavior of β-C_2_S is more advantageous for addressing the challenges posed by the low hydration activity of steel slag in practical applications.

Therefore, studying β-C_2_S is essential for gaining a better understanding of the carbonation process in the complex system of steel slag. After carbonation for 2 h, β-C_2_S can rapidly form a layered distribution structure consisting of calcite, an amorphous three-dimensional network, Si-gel, and uncarbonized β-C_2_S [17]. It was reported that compacted carbonated β-C_2_S has the highest compressive strength of 80 MPa in 24 h, and calcite and aragonite are the dominant CaCO_3_ polymorphs [16]. The high intensity of carbonated β-C_2_S is attributed to the compact stack and mechanical bond between the calcite particles, which have a large particle size and well crystallinity [18]. In addition, it is not difficult to find that previous examinations of the interactions between β-C_2_S and CO_2_ mainly focused on the carbonation reaction process, mechanical strength, and microstructure [15,16,18]. The main crystal forms of CaCO_3_ include calcite, vaterite, and aragonite, while calcite is the primary carbonation product of β-C_2_S. Vaterite and aragonite are metastable phases that can easily transform into calcite, which is the most stable crystalline phase in thermodynamics [19]. The crystallinity, crystal size, and morphology of polymorphs have significant effects on the properties of CaCO_3_ and its products [20]. Only a few studies have reported adjustments to the CaCO_3_ crystal phases in the carbonation of β-C_2_S. For example, a stabilized vaterite phase is formed in the presence of NaOH, but it reduces the carbonation degree of β-C_2_S [21]. Further studies are needed on the different crystal forms of CaCO_3_ to determine their influence.

The formation of polymorphs of CaCO_3_ is strongly affected by temperature, pH, CO_3_^2−^ concentration, and additives [19,22,23,24]. Among these CaCO_3_ polymorphs, aragonite is a high-added-value product. The aragonite crystal with a large aspect ratio easily forms whiskers, which can have a similar effect as fiber-reinforced materials. It can improve the toughness and flexural strength of cement-based materials because of the whisker–cement coalition pull-out and whisker bridging effects [25]. The addition of Mg^2+^ is the most common method used to obtain long-term stable aragonite from the carbonation of cement-based materials [5,26,27]. Furthermore, high concentrations of Mg^2+^ ions as impurity ions can promote the formation of aragonite and hinder the nucleation and growth of calcite crystals [28]. In addition, the Mg^2+^/Ca^2+^ molar ratio in the aqueous phase may be the driving force for the polymorphic changes in calcium carbonate formation [29,30]. In this study, a wet carbonation approach is proposed to synthesize highly pure needle-like aragonite from β-C_2_S as a single mineral phase. Through the study of a single mineral, the effects of temperature and the molar ratio of Mg^2+^/Ca^2+^ on the carbonation process were studied in more detail, with the aim of capturing CO_2_ and regulating the carbonation products of the β-C_2_S mineral to determine the boundary conditions under which aragonite can exist stably.

## 2. Accelerated Carbonation of β-C_2_S

**Figure 1:**
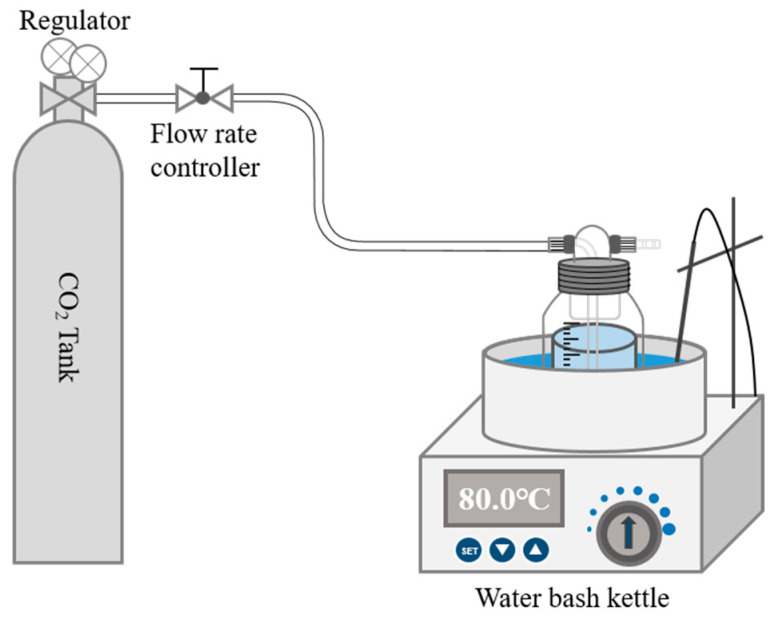
Schematic illustration of carbonation device.

According to the leaching rate of Ca^2+^ in β-C_2_S, it was measured at 460 mg/L using ICP-OES. Different Mg^2+^/Ca^2+^ molar ratios were set: 0, 0.1, 0.2, 0.5, 1.0, and 1.5. The corresponding mass of MgCl_2_ (Analytical Grade, Aladdin, Riverside, CA, USA) was weighed and dissolved into the solution. Then, β-C_2_S was carbonated at different temperatures (27 ± 0.1 °C, 40 °C, 60 °C, 70 °C, 80 °C, and 90 °C) or Mg^2+^/Ca^2+^ molar ratios in the solution for 1 h. The solid-to-solution ratio was set to 1 g/40 mL. The mixtures were immersed in a water bath and subsequently subjected to carbonation using 99% purity CO_2_ gas. The flow rate of the CO_2_ gas was controlled by an airflow meter at 80 mL/min. The carbonation device is shown in Figure 1. After the completion of the carbonation reaction, all the extraction-filtered samples were immersed in anhydrous ethanol for 24 h. Finally, the ultimate samples were obtained by drying at 50 °C for 24 h in a vacuum drying oven. Among them, the β-C_2_S powder samples with Mg^2+^/Ca^2+^ molar ratios of 0, 0.1, 0.2, 0.5, 1.0, and 1.5 after carbonation were named as M0, M0.1, M0.2, M0.5, M1.0, and M1.5, respectively.

### 2.1. Synthesis of β-C_2_S

**Figure 2:**
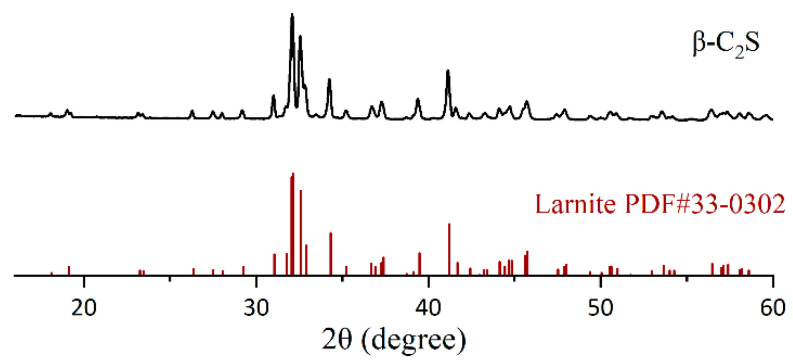
XRD pattern of the highly purified β-C_2_S.

The β-C_2_S phase was synthesized via solid-state sintering. CaCO_3_ (Analytical Grade, Aladdin) and SiO_2_ (Analytical Grade, Aladdin) were mixed at a molar Ca/Si ratio of 2:1. To stabilize the β-polymorph and inhibit γ-C_2_S formation, 0.5 wt.% B_2_O_3_ (Analytical Grade, Sinopharm, Beijing, China) was introduced during blending. Then, the mixture was placed in a drying oven at 105 °C for 24 h. The dried powder was pressed into cylinders at a pressure of 16 MPa. After sintering at 1450 °C for 3 h, the sintered cylinders were cooled down to room temperature in a rapid wind-cooling process to obtain high-purity β-C_2_S. The x-ray diffraction (XRD) pattern of the synthetic β-C_2_S phase is shown in Figure 2.

**Figure 3:**
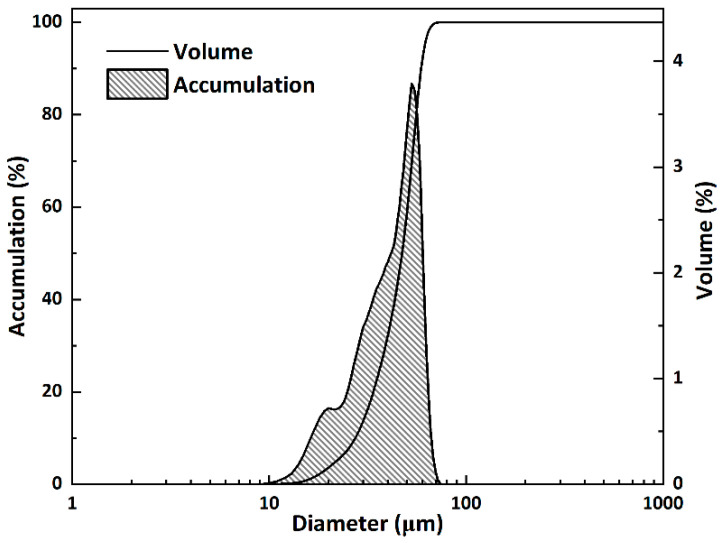
Particle size distribution of synthesized β-C_2_S powder.

The pattern matched well with the PDF 49-25256 pattern for larnite. Subsequently, the sintered block of β-C_2_S was powdered by a ball mill until most of the powder passed 160 μm. Particle size was determined using a laser diffraction particle size analyzer (NKT6200, Shandong NKT Instrumengts Co., Ltd., Jinan, China) with ethanol as a dispersant. The particle size distribution of β-C_2_S is shown in Figure 3. The particle size characteristics of the synthesized β-C_2_S, including the volume mean diameter (D_[3,4]_) and percentile values (d_10_, d_50_, d_90_), were determined as 17.68 μm, 1.61 μm, 12.27 μm, and 40.72 μm, respectively.

### 2.2. Characterization Method

#### 2.2.1. X-Ray Diffraction (XRD)

The mineral composition of the carbonated β-C_2_S samples was analyzed using X-ray diffraction (XRD, Bruker, Billerica, MA, USA, D8 Advance) with Cu Kα_1,2_ radiation (λ_1_ = 0.15406 nm, λ_2_ = 0.15444 nm) at 40 kV and 40 mA. The quantitative analysis of mineral composition was determined using the Rietveld method (QXRD). Then, 10 wt.% ZnO internal standard was homogeneously mixed with the sample to enable quantification of the crystalline and amorphous phases through Rietveld refinement-based XRD analysis. The amorphous phase content was determined as amorphous and crystalline non-quantified (ACn) [31]. MDI Jade 6.0 was used for mineral identification, and TOPAS4.2 software was utilized for the Rietveld method. The final global optimization parameters include background coefficient, cell parameters, bias error, peak shape parameters, phase fraction, and preferred orientation. The data for qualitative analysis were collected over a range of 5–80° (2θ) with a step width of 0.02° and time per step of 0.5 s.

#### 2.2.2. Thermogravimetric Analyzer (TGA)

The decomposition temperature and calcium carbonate content (including well crystalline, poorly crystalline, and amorphous phases) were determined using (TG-DSC, Mettler-Toledo, Columbus, OH, USA). The previously dried powder (20 ± 1 mg) was weighed and placed in a corundum crucible. It was then heated from 50 °C to 1000 °C at a heating rate of 10 °C/min under a nitrogen atmosphere (50 mL/min). CO_2_ sequestration capacity (g/kg) is expressed as the amount of CO_2_ sequestered by 1 kg of raw mineral. This can be calculated using Equation (1) [15], where CO_2_ (wt.%) represents the weight loss resulting from the decarbonization of CaCO_3_ as measured by thermogravimetric analysis. The temperatures ranging from 300 °C to 850 °C were selected for the decomposition of the CaCO_3_ [32].

(1) $${\mathrm{CO}}_{2}\;\mathrm{sequestration}\;\left(g/\mathrm{kg}\right)=\frac{{\mathrm{CO}}_{2}\;(\mathrm{wt}.\%)}{100-{\mathrm{CO}}_{2}\;(\mathrm{wt}.\%)}\times 1000$$

The carbonation degree of β-C_2_S (δ_CaO_, %) was calculated using Equation (2) [33], where MW_CO2_ and MW_CaO_ represent the molar masses of CO_2_ (44 g/mol) and CaO (56 g/mol), respectively. CaO_total_ is the CaO content of the raw β-C_2_S (63.3%).

(2) $$\delta \mathrm{CaO}\;\left(\%\right)=\frac{\frac{{\mathrm{CO}}_{2}\;\left(\mathrm{wt}.\%\right)}{100-{\mathrm{CO}}_{2}\;\left(\mathrm{wt}.\%\right)}\times \frac{1}{\mathrm{MW}{\mathrm{CO}}_{2}\;\left(\mathrm{kg}/\mathrm{mol}\right)}}{\mathrm{CaO}\mathrm{total}\;(\mathrm{kg}/\mathrm{kg})/\mathrm{MW}\mathrm{CaO}\;\left(\mathrm{kg}/\mathrm{mol}\right)}\times 100$$

#### 2.2.3. Fourier Transform Infrared Spectroscopy (FT-IR)

Fourier transform infrared spectroscopy (FT-IR) was recorded on a Nicolet iS10 spectrophotometer produced by Thermo Fisher Scientific (Waltham, MA, USA) using the KBr pellet technique. The scanning range was 400–4000 cm^−1^, and the spectral resolution was 4 cm^−1^. Spectroscopic software (OPUS, Version 5.5) was used to record the spectra.

#### 2.2.4. Field Emission Scanning Electron Microscopy (FE-SEM)

The morphology and size of the CaCO_3_ formed during carbonation were analyzed using field emission scanning electron microscopy (FE-SEM, FEINOVA Nano SEM 450, Hillsboro, OR, USA). The accelerating voltage of the FE-SEM was operated at 5 kV. Each sample was sputter-coated with a platinum film for 30 s. The particle size distribution of calcium carbonate in the SEM images was statistically analyzed using ImageJ (Image Processing and Analysis in Java, v1.54g) software [34]. Many regions were selected for each sample to obtain SEM images, and the number of crystal particles was sufficient for the size statistics.

#### 2.2.5. Inductively Coupled Plasma–Optical Emission Spectrometry (ICP-OES)

One g of dried β-C_2_S powder was transferred into a polyethylene (PE) bottle. Then, 40 mL of deionized water was added, and the mixture was immediately stirred at 600 rpm for 30 min using a magnetic stirrer, with the PE bottle remaining sealed throughout the process. The suspension was filtered through a vacuum filtration apparatus to separate the supernatant from the solid residue. The supernatant was diluted 10-fold with a pre-prepared 6.5% dilute HNO_3_ solution to ensure stability. The Ca^2+^ concentration in the diluted solution was determined using inductively coupled plasma optical emission spectrometry (ICP-OES, PerkinElmer Optima 2000 DV, Waltham, MA, USA).

## 3. Results and Discussion

### 3.1. Effect of Temperature on the Carbonation Process of β-C_2_S

#### 3.1.1. Development of Carbonation Products

**Figure 4:**
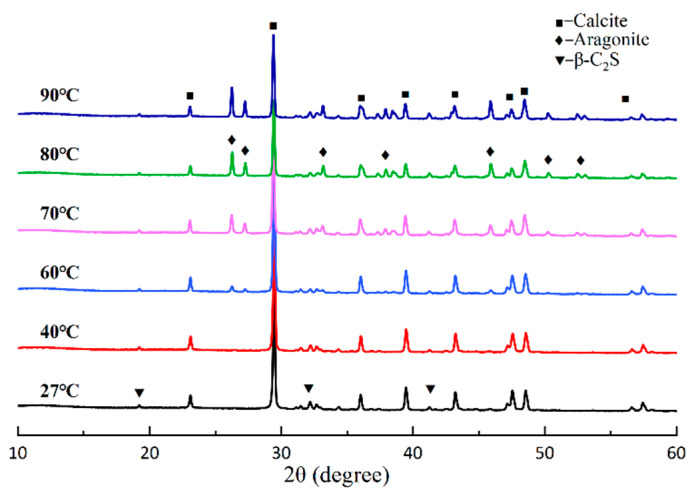
XRD pattern of β-C_2_S carbonated for 1 h at different temperatures.

**Figure 5:**
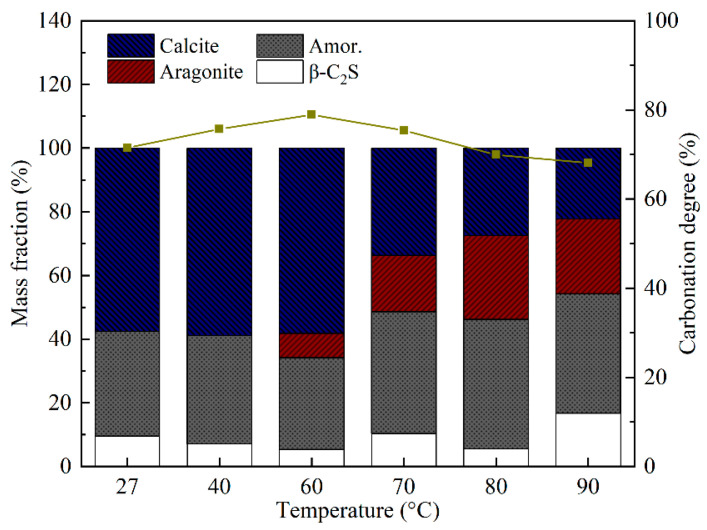
Composition of products and degree of carbonation at different temperatures.

Figure 4 and Figure 5 show the XRD results of the carbonated samples at different temperatures, along with the corresponding quantitative analysis results obtained via the Rietveld method. After carbonation at room temperature, the primary crystalline product for the pure β-C_2_S was calcite, which accounted for 57.6%. However, it was clearly seen that after the reaction temperature exceeded 60 °C, the peaks of aragonite appeared. Consistent with other reported literature, the formation of aragonite typically occurs at temperatures higher than 60 °C without any additives [35,36]. As the temperature rose from 60 °C to 90 °C, the peak height of the aragonite became more pronounced. Correspondingly, the peak height of the calcite decreased. Therefore, it could be concluded that the higher temperature promoted the formation of aragonite. Figure 2 clearly shows the relative change in calcite and aragonite content. The aragonite content reaches the maximum value of 26.3% at 80 °C. Moreover, the carbonation degree (δ_CaO_) of the β-C_2_S increased from 71.5% to 78.9% as the temperature rose to 60 °C, and then decreased to 68.1% at 90 °C. The rise in temperature increases the mass transfer rate, promotes the thermal motion of molecules, increases the average kinetic energy of molecules, and accelerates the carbonation reaction [37]. However, the effect of temperature is usually complex. Higher temperatures promote the leaching of Ca^2+^ ions and chemical reactions but reduce the solubility of CO_2_ [38]. Furthermore, since the carbonation reaction of β-C_2_S is exothermic, elevated temperature enhances the reaction kinetics within a limited range by increasing molecular collision frequency, but excessive heat may thermodynamically suppress the reaction extent due to Le Chatelier’s principle. Consequently, when the temperature was 60 °C, the CO_2_ sequestration and δ_CaO_ of the β-C_2_S were 401.79 g/kg and 78.9%, respectively, and they decreased to 357.82 g/kg and 69.9% at 80 °C, although it appears that the most suitable temperature for the carbonation reaction was 60 °C. Nevertheless, as reported by Shen et al. and You et al. [5,27], to produce needle aragonite by carbonating β-C_2_S, it is preferable to choose a temperature of 80 °C. It should be noted that no reflection of crystalline silicon dioxide was observed in any of the carbonation samples, indicating that the silicon dioxide formed during the carbonation process was amorphous [21,39]. The carbonation equation for β-C_2_S is shown in Equation (3).

(3) $$2CaO\cdot {SiO}_{2}+2{CO}_{2}\to 2{CaCO}_{3}+{SiO}_{2}\left(\mathrm{gels}\right)$$

**Figure 6:**
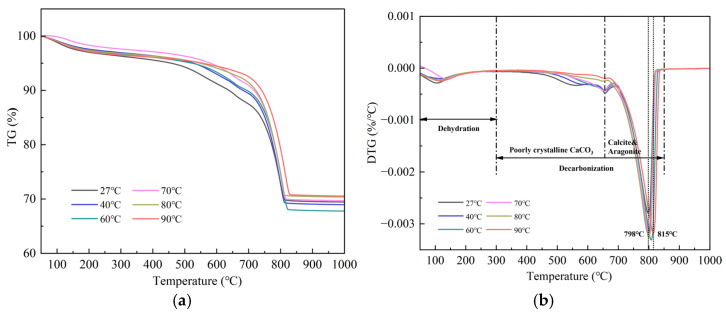
TG and DTG curves of β-C_2_S were obtained after carbonation at various temperatures. (**a**) TG curve. (**b**) DTG curve.

From the TG and DTG results in Figure 6, three distinct mass loss stages are observed: Stage I (50–300 °C): A minor mass loss attributed to the evaporation of adsorbed water and dehydration of Si-gel; Stage II (300–615 °C): A gradual mass loss (~5–8%) corresponding to the decomposition of poorly crystalline CaCO_3_, which exhibits lower thermal stability due to disordered lattice structures; Stage III (615–850 °C): A sharp mass loss (~10–15%) associated with the decarbonation of well crystalline aragonite (615–750 °C) and calcite (750–850 °C) [40]. The upward shift in the calcite decomposition peak (798 °C to 815 °C) with the increase in carbonation temperature indicates enhanced crystallinity, as slower ion migration at higher temperatures promotes ordered crystal growth. Notably, the flattening of the Stage II peak at elevated temperatures aligns with the reduced formation of poorly crystalline CaCO_3_, further supporting the dominance of stable polymorphs under high-temperature conditions.

**Figure 7:**
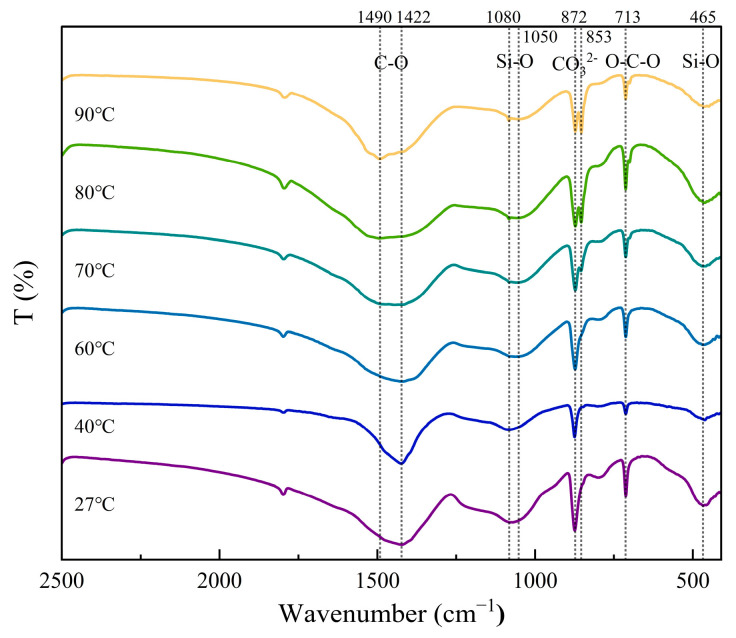
Effect of carbonation temperatures on FT-IR spectra of β-C_2_S carbonated for 1 h.

Figure 7 shows the FT-IR spectra of β-C_2_S after carbonation at different temperatures. The absorption bands at around 1080 cm^−1^ are attributed to the ν_3_ mode of the Si-O bond, characteristic of Q^3^ silicate trahedra in Si-gel [41]. Similarly, the band at ~465 cm^−1^ corresponds to the ν_4_ mode of the O-Si-O bond in Si-gel [42]. As the temperature rose from 27 °C to 80 °C, the absorption band shifted from 1080 cm^−1^ to 1050 cm^−1^. It indicated the decreased polymerization of silica-bearing materials [41]. Notably, the characteristic vibration bands of C-S-H (e.g., Si-O stretching at ~970 cm^−1^ and O-H bending at ~1640 cm^−1^ [43]) were absent in all the spectra, indicating no detectable formation of calcium silicate hydrate during carbonation. The antisymmetric C-O stretching ν_3_ bonds in calcite are at 1422 cm^−1^. The out-of-plane bending vibrations (ν_2_) of the CO_3_^2−^ bands at ~872 cm^−1^ and 853 cm^−1^ were assigned to the spectra characteristics of calcite and aragonite, respectively [26]. Furthermore, the O-C-O bending (in-plane bending vibration, ν_4_, 713 cm^−1^) band was attributed to the spectra of calcite and aragonite [43]. It should be noted that the FT-IR spectra of carbonated β-C_2_S samples showed that the characteristic absorption band of aragonite at 853 cm^−1^ intensified with increasing temperature. It could be concluded that the higher carbonation temperature has a positive effect on the formation of aragonite.

The XRD analysis revealed a significant enhancement of aragonite characteristic peaks (26.2°, 27.3°) at temperatures ≥60 °C, accompanied by a weakening of the calcite main peak (29.4°). The QXRD results revealed that the aragonite content reached 26.3% at 80 °C, suggesting that elevated temperature drives its preferential crystallization. TG-DTG analysis revealed enhanced thermal stability of calcite, as evidenced by an increase in decomposition temperature from 798 °C (at 27 °C) to 815 °C (at 90 °C). Additionally, the flattened mass loss peak in the 300–615 °C range indicated a reduction in poorly crystalline CaCO_3_ content. The SEM images exhibited well-defined slender aragonite needles (average aspect ratio: 12.09) at 80 °C, signifying suppressed disordered growth under elevated temperatures. The FT-IR spectra further supported structural ordering, as evidenced by intensified absorption bands at 853 cm^−1^ (aragonite ν_2_ mode) and 1083 cm^−1^ (CO_3_^2−^ symmetric stretching). To sum up, all the results indicated that high temperatures enhance the crystallinity of CaCO_3_ (especially aragonite) to a certain extent [4].

#### 3.1.2. Microstructural Analysis

**Figure 8:**
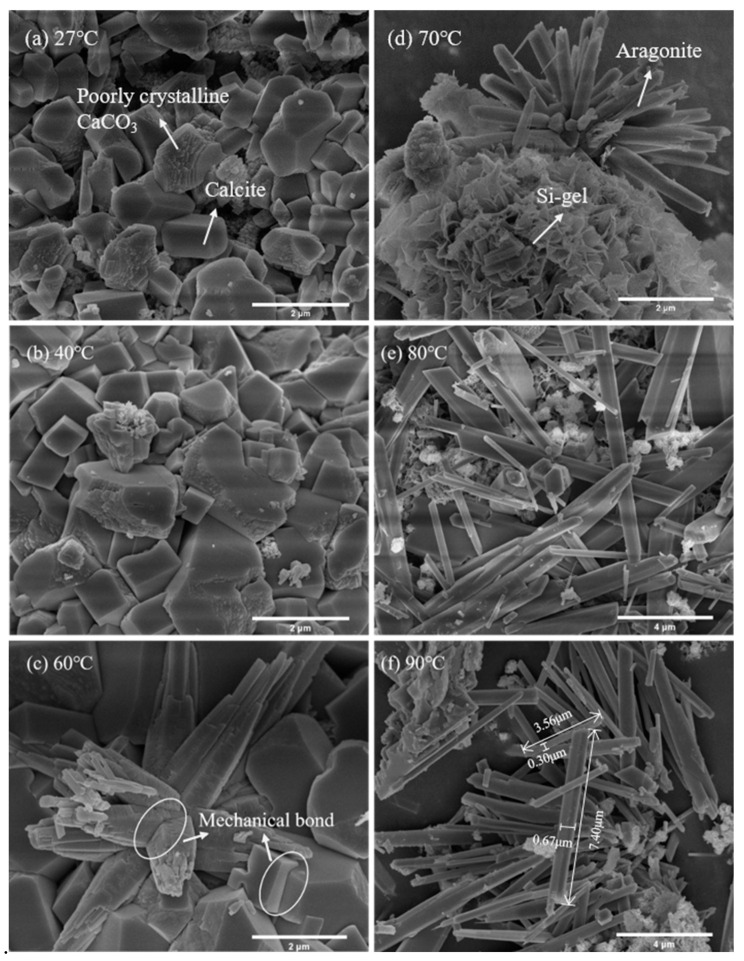
Product morphology of β-C_2_S after carbonation reaction at different temperatures.

The SEM images of the β-C_2_S after 1 h of carbonation at various temperatures are shown in Figure 8. This indicates that the temperature had a significant effect on the morphology of the carbonation products formed. Generally, calcite often exhibits rhombohedral or cubic morphologies, while aragonite typically shows fiber- or needle-like morphologies, due to their distinct crystal structures [22]. Hence, cubic calcite and needle-like aragonite can be observed in Figure 8a and 8c, respectively. However, there are signs of surface roughness and incomplete crystal growth in both calcium carbonate crystals. At the same time, it can also be observed that a large number of disordered lamellar hydration products of Si-gel are formed (Figure 8d). Nevertheless, the calcite crystals displayed a smooth surface and a regular shape at 40 °C (Figure 8b). Similarly, the aragonite crystal morphology became slenderer and regular when the temperature increased from 60 °C to 90 °C. Table 1 shows the average size of the aragonite crystals at different temperatures, as measured by the ImageJ software. The results show that the aragonite crystal grows faster and the aspect ratio increases with the temperature rise. According to Wang [44], the formation of aragonite is mainly affected by the increase in temperature because aragonite grows faster at high temperatures than calcite. It was determined that the average length and aspect ratio of the needle-like aragonite at 80 °C were ~4.77 μm and 12.09, respectively.

### 3.2. Effect of Mg^2+^/Ca^2+^ on the Carbonation Products of β-C_2_S

#### 3.2.1. Effect of Mg^2+^/Ca^2+^ on the Aragonite Formation

**Figure 9:**
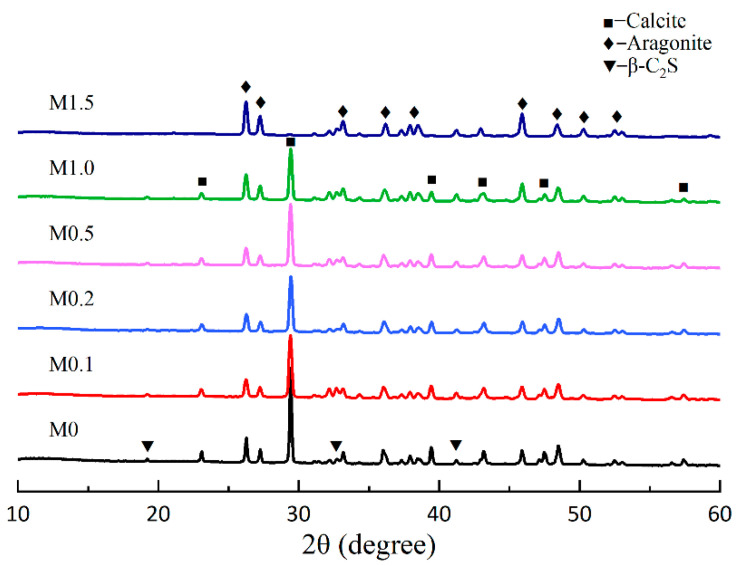
XRD pattern of β-C_2_S carbonated for 1 h in different molar ratios of Mg^2+^/Ca^2+^.

**Figure 10:**
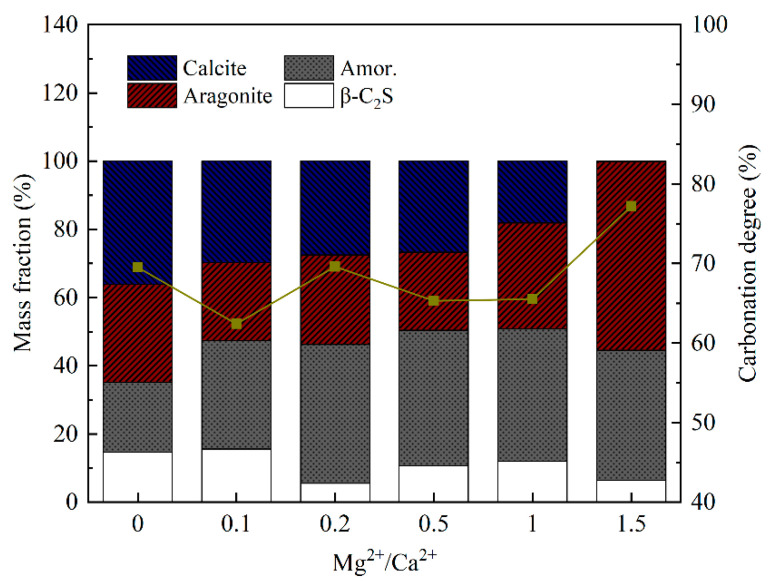
Composition of products and degree of carbonation at different molar ratios of Mg^2+^/Ca^2+^.

The effect of different Mg^2+^/Ca^2+^ ratios on the XRD patterns of β-C_2_S carbonated for 1 h at 80 °C is shown in Figure 9. The specific contents of aragonite and calcite (revealed by the Rietveld method), as well as the carbonation degree of β-C_2_S (calculated by TG analysis), are shown in Figure 10. The XRD pattern of β-C_2_S carbonated in deionized water without the MgCl_2_ addition reveals that the products consist of calcite, aragonite, an amorphous phase, and a small amount of unreacted β-C_2_S. The presence of the aragonite phase was attributed to the initial reaction temperature of 80 °C, while the calcite phase was the primary product of the carbonation. However, it can be clearly observed that the strongest characteristic peak of calcite at 29.4° gradually weakens with the increase in Mg^2+^/Ca^2+^ ratios in the solution. Furthermore, the peak disappeared when the ratio of Mg^2+^/Ca^2+^ was 1.5. In contrast, the peaks at 26.2°, 27.3°, and 45.9° became more intense, which is attributed to the characteristic peaks of aragonite. It can be seen that the concentration of Mg^2+^ has a significant effect on aragonite formation.

Figure 10 shows the specific changes in the content of the two types of calcium carbonate crystals. When the molar ratio of Mg^2+^/Ca^2+^ was 1.0, the content of the aragonite was 32.4%, while the content of the calcite was 18.6%. Aragonite became the main crystal phase of calcium carbonate. When the molar ratio of Mg^2+^/Ca^2+^ was further increased to 1.5, the aragonite content increased from 32.4% to 55.3%, while the calcite content decreased from 18.6% to 0.3%, and calcite was almost undetectable. However, it should be pointed out that the carbonation degree (δ_CaO_) of β-C_2_S exhibited a generally decreasing trend with the increasing ratio of Mg^2+^/Ca^2+^ in the solution. However, when the Mg^2+^/Ca^2+^ ratio was 1.5, the carbonation degree of the β-C_2_S increased to 77.2%. From the QXRD analysis (Figure 10), it can be concluded that aragonite was the crystalline form of calcium carbonate at this time. At lower Mg^2+^/Ca^2+^ ratios (0–1.0), Mg^2+^ ions adsorb onto the active growth sites of calcite crystals, suppressing calcite nucleation and promoting aragonite formation [28,29]. However, the competitive adsorption of Mg^2+^ also partially inhibits Ca^2+^ leaching from β-C_2_S, leading to a gradual decline in δ_CaO_. At Mg^2+^/Ca^2+^ = 1.5, the high Mg^2+^ concentration stabilizes aragonite as the dominant polymorph, which exhibits faster nucleation kinetics compared to calcite under these conditions [30]. This reduces the energy barrier for CaCO_3_ precipitation, thereby enhancing carbonation efficiency. Thermodynamic modeling by You et al. suggests that elevated Mg^2+^/Ca^2+^ ratios shift the equilibrium toward aragonite formation while destabilizing calcite. This reduces the Gibbs free energy barrier for carbonation, favoring aragonite precipitation and improving the overall reaction kinetics [27].

**Figure 11:**
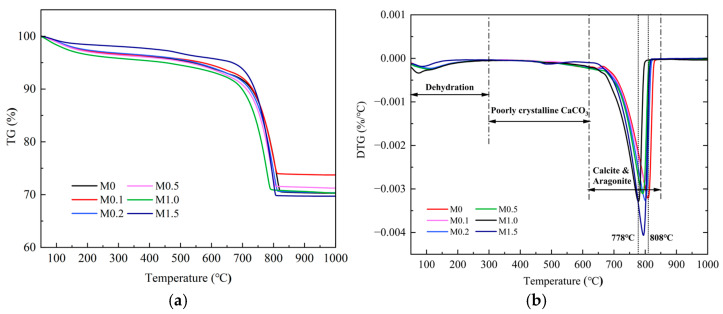
Thermal analysis of β-C_2_S carbonated for 1 h under different molar ratios of Mg^2+^/Ca^2+^. (**a**) TG curve. (**b**) DTG curve.

Figure 11 shows the results of the thermal analysis of the carbonated β-C_2_S samples at different ratios of Mg^2+^/Ca^2+^. A slight mass loss was observed in carbonated β-C_2_S samples below 300 °C, attributed to the evaporation of free water or dehydration resulting from the formation of highly polymerized silica gel [45]. The formation of poorly crystalline calcium carbonate can be confirmed by the mass loss between 300 °C and 620 °C [18]. A sharp and significant mass loss was observed above 620 °C in all the carbonated samples. The primary weight loss peak associated with the decomposition of well crystalline calcium carbonate was located at 620–850 °C. This could be due to the decomposition of carbonate, which includes calcite and aragonite [15]. According to the DTG curve, the weight loss peak for calcite was located at ~808 °C. Afterward, the weight loss peak shifted to ~778 °C as the ratio of Mg^2+^/Ca^2+^ was increased from 0 to 1.0. This phenomenon may be attributed to the presence of abundant aragonite with a lower decomposition temperature [46,47]. It is suggested that Mg^2+^ inhibits the transformation of metastable aragonite into stable calcite and promotes the stable existence of aragonite. This finding is consistent with the results of quantitative XRD analysis.

**Figure 12:**
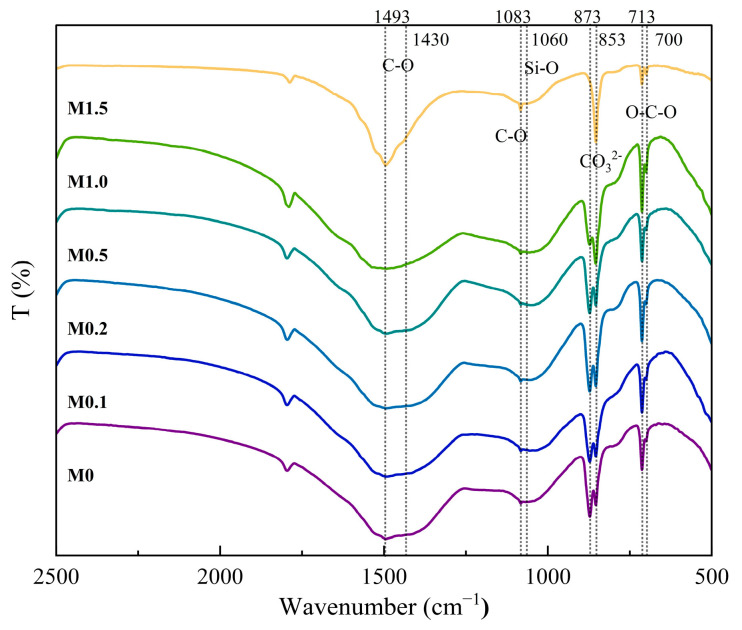
FT-IR spectrum of β-C_2_S carbonated for 1 h under different molar ratios of Mg^2+^/Ca^2+^.

To further analyze the carbonation products, FT-IR spectra of the samples carbonated for 1 h at various ratios of Mg^2+^/Ca^2+^ were conducted, and the results are shown in Figure 12. The broad band observed between 960 and 1080 cm^−1^ in the carbonated samples is linked to the calcium silicate phases [26]. It can be seen that this band was located at ~1055 cm^−1^, which was attributed to the Si-O symmetric stretching vibration in the Q^3^ units of the Si-gel [41]. No obvious changes were observed in this band during the carbonation process, indicating that Mg^2+^ would not affect the degree of polymerization of Si-gel. Usually, the in-plane bending (ν_2_), stretching vibration (ν_3_), and out-of-plane bending (ν_4_) of the C-O bond, located at 873 cm^−1^, 1430 cm^−1^, and 713 cm^−1^, respectively, were the characteristic absorption bands of calcite [43]. The symmetric stretch (ν_1_) of CO_3_^2−^ at 1083 cm^−1^ and the absorption band at 700–712 cm^−1^ (ν_4_) were attributed to the characteristic absorption bands of aragonite [26]. However, the symmetric stretching vibration (ν_1_) of CO_3_^2−^ (1083 cm^−1^) is not observed in Figure 7. This is attributed to the low aragonite content (26.3% at 80 °C) in samples carbonated across varying temperatures (27–90 °C). Additionally, the symmetric stretching vibration (ν_1_) of CO_3_^2−^ in aragonite (1083 cm^−1^) and the ν_3_ antisymmetric stretching vibration (ν_3_) of Si–O bonds in silica gel (1080 cm^−1^) exhibit close wavenumber proximity (Δ ≈ 3 cm^−1^). Consequently, the weak ν_1_ signal of aragonite is obscured easily by the broad Si–O vibrational band, resulting in a single unresolved broad peak in the FT-IR spectra [26]. In contrast, Figure 12 corresponds to carbonated samples with varying Mg^2+^/Ca^2+^ molar ratios. The aragonite content is significantly increased to 55.3% (Mg^2+^/Ca^2+^ = 1.5), and the enhanced intensity of the symmetric stretching vibration (ν_1_, 1083 cm^−1^) induces a peak splitting phenomenon at 1080 cm^−1^, enabling a clear distinction between the overlapping silica gel and aragonite signals [26].

It was obvious that the intensity of the narrow bands, located at around 853 cm^−1^ and 1083 cm^−1^, exhibited a sharp increase with the increase in ratios of Mg^2+^/Ca^2+^ due to the formation of the aragonite phase. In particular, the broad absorption band located at ~1493 cm^−1^ (ν_3_) became narrower with the increase in ratios of Mg^2+^/Ca^2+^, indicating that aragonite became the primary calcium carbonate phase [43]. Hence, based on the results of XRD and FT-IR, when the molar ratio of Mg^2+^/Ca^2+^ was 1.5, the conversion of aragonite to calcite can be inhibited, leading to the formation of high-purity aragonite.

#### 3.2.2. Microstructure Analysis

**Figure 13:**
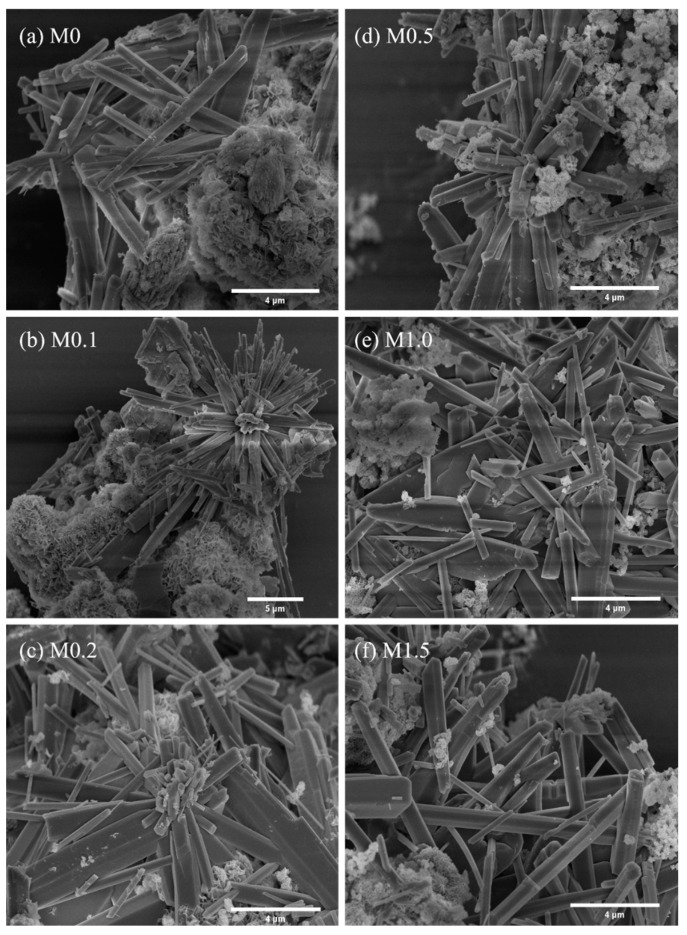
Effect of different Mg^2+^/Ca^2+^ molar ratios on the morphology of the products after carbonation of β-C_2_S.

The morphology of the carbonated products after 1 h at various ratios of Mg^2+^/Ca^2+^ in the solution is shown in Figure 13. Needle-like aragonite was observed under all conditions, attributed to the combined effects of 80 °C and Mg^2+^. Similarly, the average length and diameter of aragonite crystals were measured under various molar ratios of Mg^2+^/Ca^2+^ using ImageJ software, as shown in Table 2. The average length and diameter of these aragonite crystals were 3–8 μm and ~1 μm, respectively. Furthermore, the aspect ratio of aragonite decreased from 13.22 to 9.39 as the molar ratio of Mg^2+^/Ca^2+^ increased from 0 to 1.5. This indicated that excessive Mg^2+^ should be avoided in the synthesis of needle-like aragonite with a high aspect ratio. In addition, it can be observed that the aragonite (Figure 13b,c) was not tightly packed together like the calcite (Figure 8a,b), but rather radial or cluttered, crossing each other. It illustrated that the needle-like aragonite tends to be separated individually. To be brief, the results of XRD, TG, FT-IR, and SEM show that the presence of Mg^2+^ promotes the formation of needle-like aragonite. When the molar ratio of Mg^2+^/Ca^2+^ was 1.0, the content of aragonite reached 31.0%, and aragonite became the primary crystal form of calcium carbonate.

After preparing the β-C_2_S into test blocks, we conducted a 72 h carbonation experiment and performed compressive and flexural strength tests in accordance with the GB/T 17671-2021 [48]. As shown in Figure S1a, the carbonated β-C_2_S specimens exhibited compressive strengths ranging from 100 to 111.4 MPa, which are significantly higher than the 28-day compressive strength of ordinary Portland cement (OPC, typically 40–50 MPa). Similar results were reported by Mu et al., who observed a compressive strength of 80 MPa in carbonated β-C_2_S-aragonite composites [16].

Furthermore, Figures S1b and S2b demonstrate that the presence of aragonite preferentially enhances the flexural strength of β-C_2_S compared to its compressive strength. The flexural strength progressively increases with higher synthesized aragonite content, attaining a maximum value of 31.6 MPa (Figure S2a), which corresponds to a 38.5% improvement relative to the reference sample. This performance substantially exceeds the typical flexural strength range of ordinary Portland cement (OPC, 7–10 MPa). Cao et al. attributed the 30–40% improvement in flexural strength of mortar to the fiber-reinforcing effect of aragonite and the dense microstructure of calcium carbonate [49]. Therefore, the carbonated β-C_2_S composites have the potential to replace traditional cement in terms of mechanical properties.

## 4. Conclusions

In this study, a wet carbonation method was developed to produce needle-like aragonite using β-C_2_S. The effects of temperature and the Mg^2+^/Ca^2+^ molar ratio in the solution on the behavior of carbonated β-C_2_S were investigated. Based on all the experimental results above, two conclusions can be drawn.

(1) The needle-like aragonite, having an average length of 1–6 μm and an average aspect ratio of 6–13, can be quickly synthesized when the temperature of carbonation increases from 60 °C to 90 °C. Although 60 °C was the optimal temperature for enhancing the carbonation degree of β-C_2_S, the most appropriate temperature for producing aragonite with a high aspect ratio was 80 °C. At this temperature, the carbon sequestration capacity of β-C_2_S was 357.82 g/kg.
(2) Besides carbonation temperature, the molar ratio of Mg^2+^/Ca^2+^ was also a crucial factor influencing the formation of needle-like aragonite. The combined effect of these two factors promoted the formation of aragonite. Mg^2+^ promoted the formation of aragonite and hindered the transformation of aragonite to calcite at the reaction temperature of 80 °C. Aragonite became the primary crystal form of calcium carbonate when the Mg^2+^/Ca^2+^ molar ratio was above 1.0. Under the condition of a Mg^2+^/Ca^2+^ molar ratio of 1.5, the content of aragonite was 55.2%, while calcite was almost non-existent, and calcium carbonate existed as aragonite crystals.

## Figures and Tables

**Table 1:** The statistical size information of aragonite particles in β-C_2_S carbonated for 1 h at different temperatures (60 °C, 70 °C, 80 °C, and 90 °C).

Temperature/°C	Length/μm	Diameter/μm	Aspect Ratio
60	1.29	0.19	6.91
70	3.49	0.44	7.98
80	4.77	0.39	12.09
90	5.67	0.45	12.60

**Table 2:** The statistical size information of aragonite particles in β-C_2_S carbonated for 1 h at different molar ratios of Mg^2+^/Ca^2+^.

Sample	Length/μm	Diameter/μm	Aspect Ratio
M0	7.80	0.59	13.22
M0.1	3.44	0.30	11.47
M0.2	5.15	0.44	11.70
M0.5	4.25	0.40	10.63
M1.0	4.86	0.46	10.56
M1.5	3.96	0.43	9.39

## Data Availability

The original contributions presented in this study are included in the article/Supplementary Material. Further inquiries can be directed to the corresponding author.

## Supplementary Materials

The following supporting information can be downloaded at: https://www.mdpi.com/article/10.3390/ma18102232/s1, Figure S1: β-C_2_S carbonated at different MgCl_2_ concentrations: (a) Compressive strength; (b) Phase composition. Figure S2: β-C_2_S carbonated at different MgCl_2_ concentrations: (a) Flexural strength; (b) Phase composition.
