# Supplementary material for: Effect of Magnesium and Temperature on the Accelerated Carbonation Progress of β-Dicalcium Silicate
Source: Materials (Basel). 2025 May 12;18(10):2232. doi: 10.3390/ma18102232 (PMC12113067; doi:10.3390/ma18102232)
Supplement: Supplementary file 1 [file materials-18-02232-s001.zip › materials-3602208-supplementary.pdf]

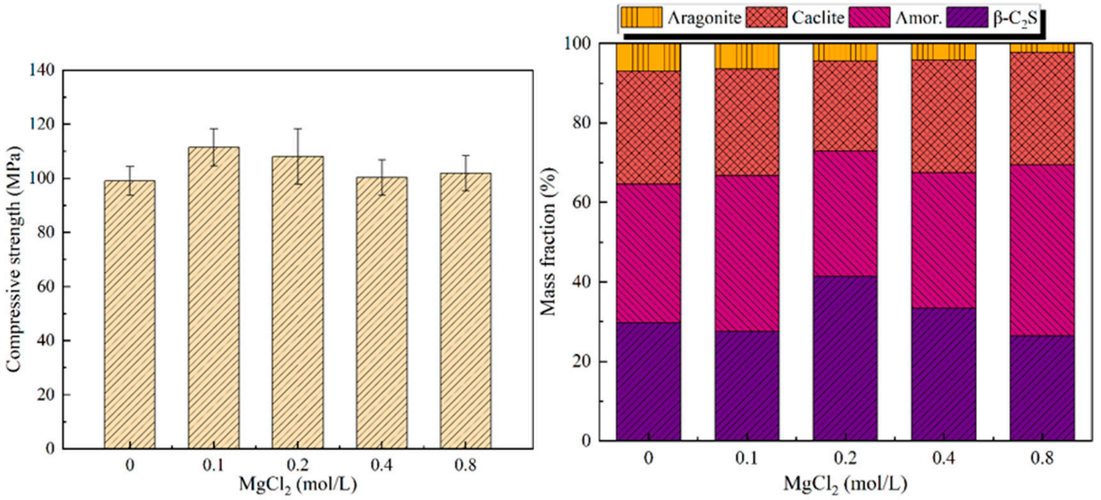

(a) Compressive strength (b) Phase composition

Figure S1. β-C_2S carbonated at different $MgCl_2$ concentrations: (a) Compressive strength; (b) Phase composition.

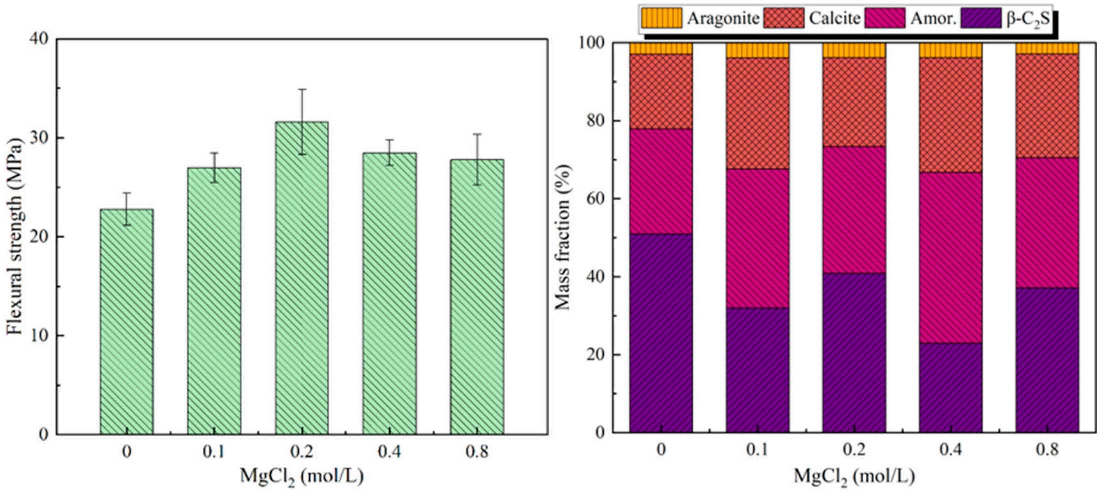

(a) Flexural strength (b) Phase composition

Figure S2. β-C_2S carbonated at different $MgCl_2$ concentrations: (a) Flexural strength; (b) Phase composition.
